# Association between intra-operative hemodynamic changes and corrective procedures during posterior spinal fusion in adolescent patients with scoliosis: A case-control study

**DOI:** 10.1097/MD.0000000000028324

**Published:** 2021-12-30

**Authors:** Kanichiro Wada, Gentaro Kumagai, Hitoshi Kudo, Sunao Tanaka, Toru Asari, Yuki Fjita, Yasuyuki Ishibashi

**Affiliations:** Department of Orthopaedic Surgery, Hirosaki University Graduate School of Medicine, Hirosaki, Aomori, Japan.

**Keywords:** corrective procedure, hypotension, posterior spinal fusion, scoliosis, thoracic cage deformity

## Abstract

Previous reports indicated that a decrease in intra-operative cardiac output and mean arterial pressure occurs due to thoracic cage deformities when patients with scoliosis are placed in the prone position. The aims of this study were to investigate the occurrence of hypotension during posterior spinal fusion in adolescent patients with scoliosis, and the association between hypotension, surgical procedures, changes of thoracic cage morphology.

This retrospective, single-center, case-control study included 106 patients who underwent surgeries for spinal deformity at our institute between June 2014 and March 2020. The inclusion criteria were: age ≤19 years at the time of surgery, lowest instrumented vertebra over L5, posterior spinal fusion as the first surgery for scoliosis, and no severe cardiac or pulmonary disease pre-operatively. Finally, 49 patients met the criteria, and were divided to 3 groups as follows: thoracic constructive curve using a 6.0-mm cobalt-chromium alloy circular rod (T-C group; n = 28); thoracolumbar/lumbar constructive curve using a 6.0-mm cobalt-chromium alloy circular rod (L-C group; n = 8); and thoracic constructive curve using a 5.5-mm cobalt-chromium alloy beam-like rod (T-B group; n = 13). The beam-like rod is characteristic as the rod is mounted to screw heads without cantilever force. Intra-operative changes in circulation associated with corrective procedures, perioperative data, and sagittal depth and sternum deviation of thoracic cage were compared between the 3 groups.

The T-C group had a higher rate of hypotension alarm than did the other groups (7 vs 0 vs 0; *P* = .047). Corrective procedures included rodding 4 times, rod rotation maneuver once, and direct vertebral rotation twice. Blood pressure was increased by pausing the correction procedures, increasing infusion, and administering vasopressors. The T-C and T-B groups had greater sternum deviation parameters than the L-C group, both before and after surgery. All parameters associated with sagittal depth and sternum deviation decreased significantly after surgery in the T-C and the T-B groups.

In corrective surgery for constructive thoracic scoliosis, the corrective procedures requiring the application of compression force in the forward direction should be closely monitored in view of their possible influence on circulatory conditions.

## 1 Introduction

Adolescent scoliosis is a 3-dimensional deformity of the thoracic cage involving vertebral translation, vertebral rotation, and rib deformities.^[1]^ Thoracic cage deformity is associated with health disturbances including pulmonary dysfunction,[^2^,^3^] back pain,^[4]^ and changes in self-image^[5]^ that can be improved by posterior spinal fusion (PSF). Furthermore, PSF also influences intra-operative circulation. Studies have reported that the prone position induces a decrease in cardiac output and mean arterial pressure in patients with scoliosis.[^6^,^7^] Hypotension during scoliosis surgery may also impair the maintenance of general anesthesia. In addition, intra-operative neuromonitoring,[^8^,^9^] a standard procedure for the prevention of spinal cord injury in scoliosis surgery, may be difficult in the presence of unstable circulation.

Thoracic cage deformity might affect the cardiopulmonary function of patients with spinal deformity during PSF because of the morphological changes in the thoracic cage during corrective procedures. However, the incidence of hypotension and the relationship between hemodynamic changes and corrective procedures in adolescent patients with scoliosis are unclear. The first aims of this study were to investigate the rate of occurrence of hypotension during posterior corrective surgery in adolescent patients with scoliosis, to clarify the association between hypotension and surgical procedures. The second aim was to clarify the difference in change of spinal and thorax cage deformities between the classes of curves and surgical procedures for considering the effect of correction of scoliosis on thoracic cage morphology.

## 2 Methods

### 2.1 Study design

This study was a retrospective, single-center, case-control study.

### 2.2 Setting and sampling technique

A total of 106 consecutive scoliosis patients who underwent spinal corrective surgeries at a single institution between June 2014 and March 2020 were potentially eligible for this study. The inclusion criteria for this retrospective case-control study were: age ≤19 years at the time of surgery, lowest instrumented vertebra over L5, PSF as the first surgery for scoliosis, and no severe cardiac or pulmonary disease before surgery. Congenital scoliosis, atypical curve, growth sparing surgery, anterior fusion, atypical surgical procedures (e.g., in situ fusion under halo-femoral traction, tandem connection of 2 rods), and lack of clinical data were excluded.

### 2.3 Participant

**Figure 1:**
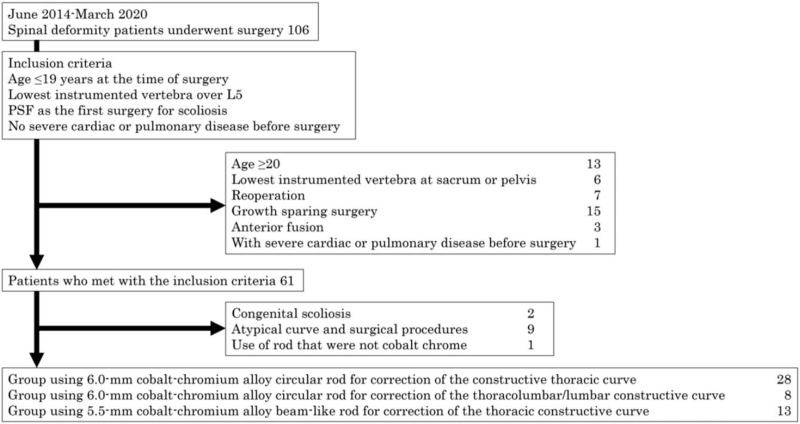
Flow chart of the selection of analyzed participants. Forty-nine patients who were analyzed in this study, were selected from 106 patients who underwent surgeries for spinal deformity between March 2014 and June 2020.

In the analysis of this study, calculated effect size was 0.108 and power was 0.647. Sixty-one adolescent patients with scoliosis met the inclusion criteria, and of these 61 patients, 1 patient with congenital scoliosis, 9 patients with atypical curves and surgical procedures, 1 patient in whom a rod that was not cobalt chrome was used, were all excluded from the study. Finally, 49 patients (mean age at time of surgery, 14.7 ± 1.9 years) were selected and their data were analyzed (Fig. 1). There were no patients whose clinical data were lacked. The study protocol was approved by the ethics committee of Hirosaki University (approval number 2019-1038). Informed consent was obtained from all the study participants.

### 2.4 Hemodynamic evaluation and definition of hypotension alarm

The primary outcome of this study was the alarm for hypotension from the anesthesiologists. Intravenous anesthesia consisted of propofol, remifentanil, and vecuronium for induction, followed by propofol, remifentanil, and vecuronium for maintenance. Anesthesiologists continuously monitored the intra-operative blood pressure using arterial catheterization. The alarm for hypotension in this study was defined as the event of decreased blood pressure that impaired maintenance of general anesthesia or when the patient's blood pressure could not be measured; such instances, the anesthesiologists informed the orthopedic surgeons. When there was an alarm, the surgeons stopped the procedure and released the correction, and the anesthesiologists increased infusion, administered vasopressors, and provided treatment where necessary. Surgeries were restarted when stable circulation was recovered. Anesthesiologists prospectively recorded data related to the alarms, including blood pressure, surgical procedures, treatments, and responses. The anesthesiologists also recorded the minimum blood pressure during each procedure, and the values were used to calculate the mean arterial pressure. The anesthesiologists monitored the patients’ hemoglobin concentration, platelet counts, and other blood data as appropriate; there was no case where tranexamic acid was used in this study.

### 2.5 Computed tomography measurements of the thoracic cage

**Figure 2:**
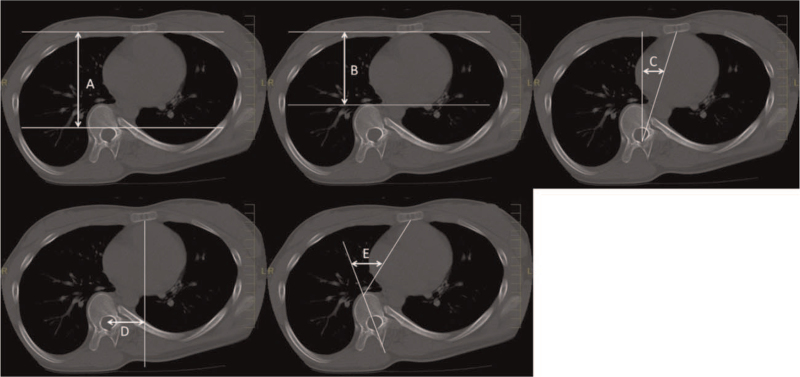
Computed tomography measurement of the thoracic cage parameters at the level of the apical thoracic vertebra. The sagittal diameter, A, is the distance between the posterior midpoint of the sternum and the anterior point of the foramen along the anteroposterior plane. The sternovertebral distance, B, is the distance between the posterior midpoint of the sternum and the anterior point of the apical vertebra along the anteroposterior plane. The midline deviation, C, is the angle between the anteroposterior plane and the line through the posterior aspect of the foramen and the anterior midpoint of the sternum. The vertebral translation, D, is the distance between the anteroposterior planes through the center of the foramen and bisecting the sternum. The thoracic rotation, E, is the angle between the plane bisecting the sternum and the anterior aspect of the vertebral body and the plane bisecting the vertebrae.

The secondary outcome was the computed tomography (CT) measurements of thoracic cage deformities. Thoracic deformity parameters including sagittal diameter,^[10]^ sternovertebral distance,^[11]^ midline deviation,^[10]^ thoracic rotation,^[12]^ and vertebral translation^[1]^ were evaluated using CT pre-operatively (Fig. 2) and the day after surgery. CT images were examined for surgical planning and to identify complications, such as screw perforation, hemothorax, and pneumothorax. The sagittal diameter and sternovertebral distance were measured to evaluate sagittal depth, while the other 3 parameters were measured to evaluate sternum deviation as a deformity feature.^[13]^ All radiographic measurements were performed on an intra-hospital computer workstation.

### 2.6 Surgical procedures

We used 6.0-mm cobalt-chrome rods for patients with main thoracic curve consistently from 2014 to 2017 and changed to 5.5-mm cobalt-chrome beam-like rods^[14]^ from January 2018. The beam-like rod is characteristic as mounting the rod to screw heads does not require cantilever force. We also used 6.0-mm cobalt-chrome rods for thoracolumbar/lumbar scoliosis continuously from 2014 to 2020.

All surgeries were performed by a single surgeon (WK) using 2 types of instruments: a 6.0-mm cobalt-chrome circle rod system and a 5.5-mm cobalt-chrome beam-like rod system. All patients were placed in the prone position on a Jackson table; facetectomies were performed through a posterior approach using midline incisions, and pedicle screws with uniplanar or multiaxial heads were chosen based on the screw insertion points. The pedicle screws were inserted into every vertebra except those without cancellous pedicles and those with obvious perforations on the pedicles during surgery. Sublaminar tape and hooks were added if needed. The auto-local bone of the spinous process and artificial bone grafts were used for all patients.

**Figure 3:**
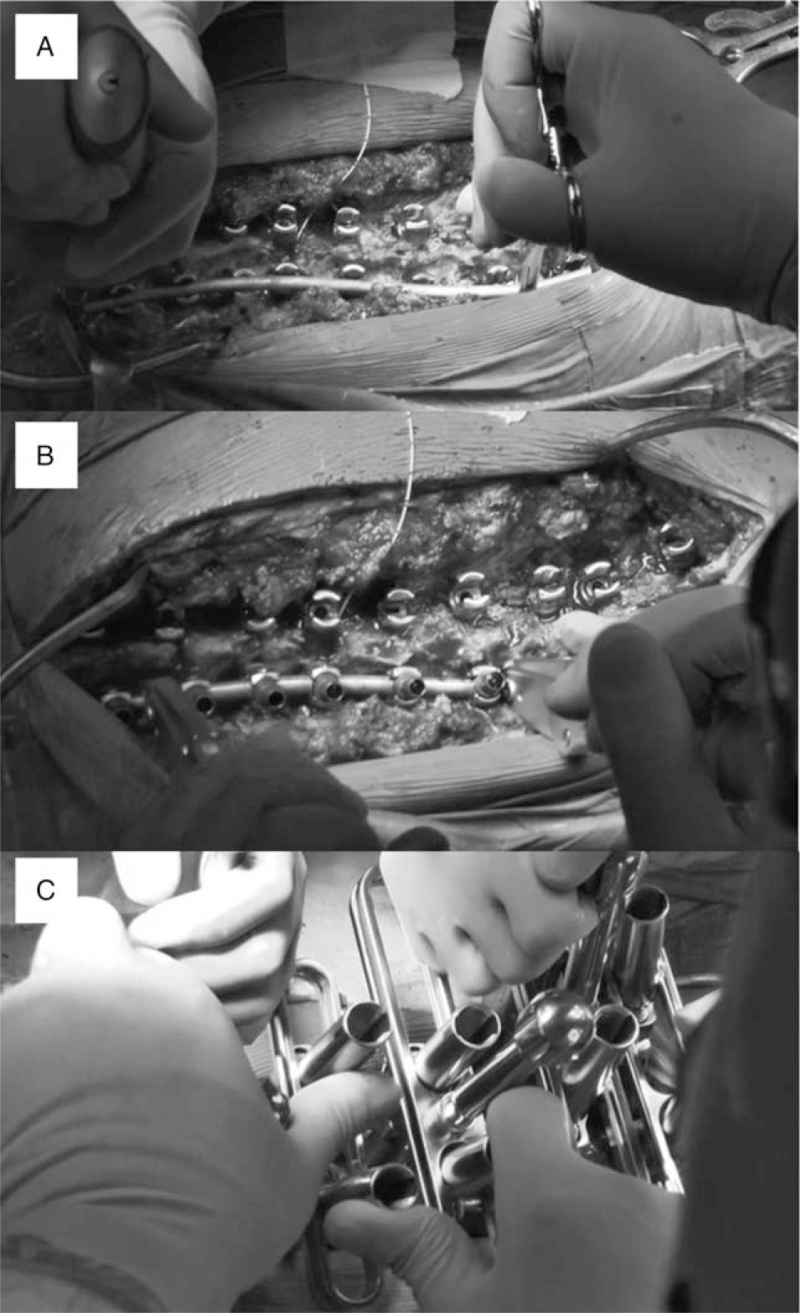
Surgical corrective procedures using pedicle screws and 6.0 mm cobalt chrome circle rod for thoracic scoliosis. A was attachment of rod with cantilever force, B was rod rotation maneuver, and C was direct vertebral rotation.

**Figure 4:**
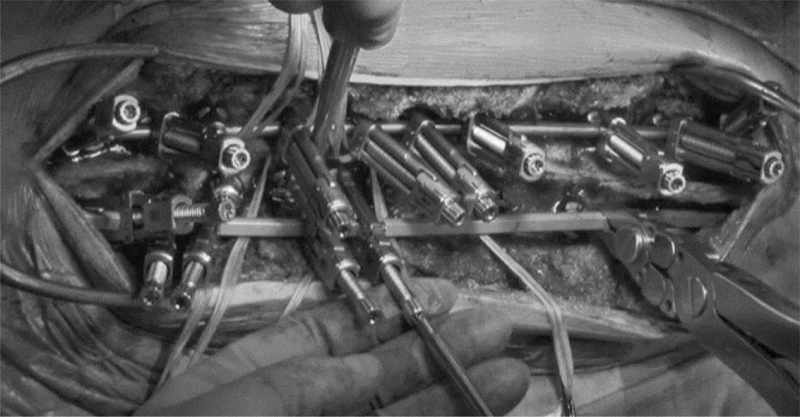
Surgical corrective procedures using pedicle screws and 5.5 mm beam-like rod for thoracic scoliosis. Rods were attached to pedicle screws using cricket-like rod fixators.

Surgical procedures were selected based on the level of the main curve and rod type. Using a 6.0-mm circular rod system in the main thoracic curve, the concave-side rod was first mounted on the proximal and distal foundations using the cantilever technique, followed by translation with the rod rotation maneuver (RRM) and direct vertebral rotation (DVR) (Fig. 3). Using the same rod system in the main thoracolumbar/lumbar curve, the convex-side rod was mounted first, and the same procedures were performed in patients with a main thoracic curve. Using a 5.5-mm beam-like rod system in the main thoracic curve, a convex round rod was inserted into screws using cricket-like rod fixators (Fig. 4). Afterward, a concave beam-like rod was mounted using rod fixators. The rods were inserted by gradually tightening the fixators, starting with the concave fixator, and followed by the one on the convex side. After fully tightening the rod fixators and partially locking the screw heads, DVR and final locking were performed. Pedicle screws were inserted using fluoroscopy or intra-operative CT-based navigation. All surgeries were performed using intra-operative neuromonitoring.

### 2.7 Clinical and respiratory evaluations

Past histories of cardiovascular, respiratory and thorax diseases, and pre-operative respiratory function including percentage of vital capacity (%VC) and forced expiratory volume in 1 second percentage (FEV1.0%) were collected from the patients’ medical records. Operative time, blood loss, usage of cell saver, number of fused vertebrae were also collected from the records.

### 2.8 Plain radiographic measurements of the spine

**Figure 5:**
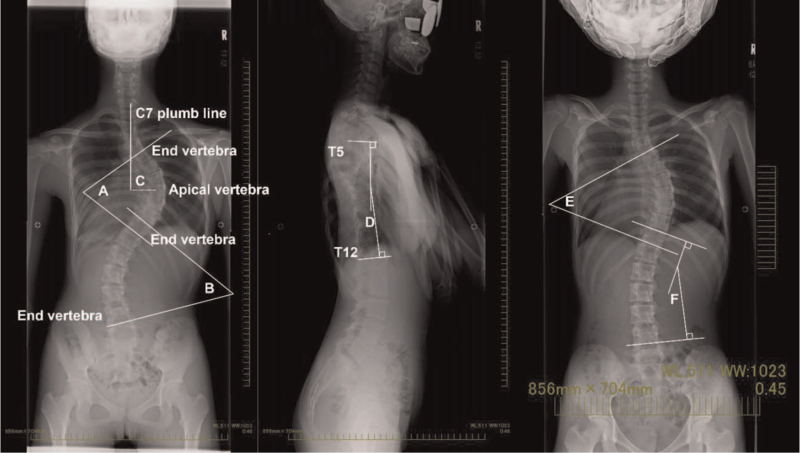
X-ray measurements of coronal and sagittal parameters. The Cobb angles, A and B, are angles between the upper and lower end vertebra of each thoracic and lumber curvature. The apical vertebra translation of thoracic curvature, C, is the distance between the midpoint of the apical vertebra and C7 plumb line. The thoracic kyphosis, D, is the angle between upper endplate of T5 and lower endplate of T12. The Cobb angles with manual traction in supine position, E and F, are the angles between upper and lower endplates of end vertebra of each thoracic and lumber curvature.

Standing anteroposterior spine plain radiographs in a relaxed neutral position and lateral radiographs with fists resting on the clavicles were taken pre-operatively and at 1 or 2 weeks postoperatively. The Cobb angle, apical vertebral translation (AVT), and thoracic kyphosis were measured using radiography. Manual traction radiographs in the supine position were performed to assess pre-operative flexibility, which was calculated by dividing the difference between the traction and standing Cobb angles by the standing angle. The corrective rate was calculated by dividing the difference between the pre-operative and postoperative standing Cobb angles by the pre-operative angle (Fig. 5).

### 2.9 Statistical analysis

The patients were divided into 3 groups according to the rod used for correction and the levels of the main curve: the 6.0-mm cobalt-chromium alloy circular rod for the corrective thoracic curve (T-C group, n = 28); thoracolumbar/lumbar corrective curve groups (L-C group, n = 8); and 5.5-mm cobalt-chromium alloy beam-like rod for the thoracic corrective curve group (T-B group, n = 13). Incidence of hypotension alarm was compared among the 3 groups. And thoracic cage parameters on CT were compared between before and after surgeries in each group. Categorical variables including sex, scoliosis classification, and hypotension alarm were compared using the chi-square test. Age, body mass index, radiographic measurements, operative time, blood loss, number of instrumented vertebrae, and mean arterial pressure were compared using the Kruskal-Wallis test. The Cobb angles on the X-ray and thoracic cage parameters on CT were compared before and after surgery in each group using the Wilcoxon test. SPSS version 22 (IBM Corp., Armonk, NY) was used for these analyses, and a value of *P* < .05 was considered statistically significant.

## 3 Results

### 3.1 Baseline characteristics according to curve patterns and corrective procedures

Age, sex, classification of scoliosis, and body mass index were not different between 3 groups. There were 3 patients with past histories of cardiovascular diseases in T-C group, none in T-L group, and 1 patient in the T-B group. Regarding respiratory/thorax diseases, there were 2, 0, and 1 patient in the T-C group, T-L group, and the T-B group, respectively. There were no significant differences in past histories. Findings of respiratory examinations, such as %VC and FEV1% were not significantly different between the 3 groups (Table 1).

**Table 1:** Clinical characteristics according–curve patterns and corrective procedures.

	T-C group (n = 28)		L-C group (n = 8)		T-B group (n = 13)		*P* value
		95%CI		95%CI		95%CI	
Age (yrs)	14.3 ± 2.1	14.3–14.4	15.4 ± 1.3	15.4–15.4	15.0 ± 1.8	15.0–15.0	.299^∗^
Sex (male/female)	5/23		0/8		1/13		.335^†^
Idiopathic/syndromic	14/7		8/0		12/1		.149^†^
BMI	20.2 ± 3.6	20.1–20.2	20.3 ± 1.8	20.2–20.3	18.9 ± 2.4	18.9–19.0	.405^∗^
Past medical histories							
Cardiovascular disease (%)	3 (10.7)		0 (0)		1 (7.7)		.619^†^
Respiratory/thorax disease (%)	2 (7.1)		0 (0)		1 (7.7)		.730^†^
%VC	85.2 ± 15.6	79.4–91.0	89.6 ± 12.1	81.2–97.9	81.6 ± 15.3	73.3–89.9	.474^∗^
FEV1.0%	79.4 ± 14.8	73.9–84.9	90.2 ± 3.9	87.5–92.9	81.3 ± 14.0	73.7–88.9	.171^∗^

### 3.2 Hypotension alarm and associated procedures

The intra-operative circulation parameters are presented in Table 2. The T-C group had a higher rate of alarm for hypotension than the other groups (7 vs 0 vs 0; *P* = .047). The corrective procedures associated with hypotension alarms were rodding in 4, RRM in 1, and DVR in 2 cases. Blood pressure eventually increased in all events by pausing and releasing the correction procedures, increasing infusion, and administering vasopressors. No neurological complications were observed postoperatively. The mean arterial pressures differed significantly across the 3 groups at the DVR and at the end of surgery. The %VC was 89.1 ± 23.1 and FEV1% was 87.9 ± 11.1 in these 7 patients that had hypotension alarm intra-operatively.

**Table 2:** Alarm of hypotension and mean arterial pressure during surgery.

	T-C group (n = 28)		L-C group (n = 8)		T-B group (n = 13)		*P* value
		95% CI		95% CI		95% CI	
Alarm of hypotension							
Number	7 (33.3)		0		0		.047^∗^
Timing							
Rodding	4						
RRM	1						
DVR	2						
Treatment							
Vasopressor	2						
Increasing infusion	2						
Release/procedure change	3						
Mean arterial pressure (mm Hg)							
Pre-skin incision	68.7 ± 9.5	68.6–68.8	75.5 ± 11.2	75.2–75.8	67.3 ± 8.7	67.2–67.5	.162^†^
Rodding	61.1 ± 11.7	60.9–61.2	68.7 ± 8.6	68.5–68.9	61.7 ± 8.7	61.5–61.8	.197^†^
RRM	60.8 ± 15.9	60.6–61.0	68.0 ± 8.2	67.8–68.2			.183^†^
DVR	59.3 ± 12.9	59.1–59.5	67.3 ± 6.9	67.2–67.5	55.5 ± 8.4	55.4–55.7	.035^†^
Final	64.1 ± 7.1	64.0–64.1	74.3 ± 5.5	74.2–74.5	63.2 ± 8.5	63.1–63.4	.006^†^

Cobb angle of the thoracic curve were 60.7 ± 21.2 degrees before surgery, 39.2 ± 23.2 degrees in pre-operative traction, and 23.3 ± 10.0 degrees postoperatively in the 7 patients that has hypotension alarms. Sagittal diameter was 100.2 ± 19.8 mm pre-operatively and 90.1 ± 14.0 mm postoperatively, sternovertebral distance was 77.7 ± 19.0 mm and 67.0 ± 13.9 mm, midline deviation was 14.4 ± 6.5 degrees and 11.2 ± 5.4 degrees, vertebral translation was 33.2 ± 18.0 mm and 24.4 ± 14.3 mm, thoracic rotation was 37.8 ± 17.8 degrees and 31.0 ± 18.7 degrees in these 7 patients, respectively. The radiographic parameters of the patients with hypotension alarm were similar to that thoracic scoliosis patients without hypotension alarm.

### 3.3 Thoracic cage deformities on CT

The thoracic cage parameters measured on CT images are shown in Table 3. Sagittal diameter and sternovertebral distance were not significantly different among the 3 groups. The T-C and T-B groups had greater sternum deviation parameters (midline deviation, thoracic rotation, and vertebral translation) than the L-C group, both before and after surgery.

**Table 3:** Computed tomography measurement of the thoracic cage.

	T-C group (n = 28)		L-C group (n = 8)		T-B group (n = 13)		*P* value
		95% CI		95% CI		95% CI	
Sagittal diameter (mm)							
Pre-operative	100.7 ± 15.7	100.5–100.9	101.5 ± 9.7	101.3–101.7	90.4 ± 17.0	90.1–90.7	.197^∗^
Postoperative	92.5 ± 11.4	92.4–92.6	97.6 ± 12.5	97.3–97.9	83.1 ± 14.0	82.9–83.4	.070^∗^
Sternovertebral distance (mm)							
Pre-operative	78.1 ± 15.1	77.9–78.3	79.2 ± 9.9	79.0–79.4	68.6 ± 15.9	68.4–68.9	.238^∗^
Postoperative	70.0 ± 11.2	69.9–70.2	74.9 ± 11.9	74.6–75.2	61.1 ± 12.9	60.9–61.4	.073^∗^
Midline deviation (degrees)							
Pre-operative	15.2 ± 5.9	15.1–15.3	5.1 ± 2.8	5.1–5.2	16.9 ± 4.8	16.9–17.0	<.001^∗^
Postoperative	11.1 ± 5.4	11.1–11.2	3.6 ± 3.6	3.5–3.7	13.2 ± 3.6	13.1–13.3	<.001^∗^
Vertebral translation (mm)							
Pre-operative	34.4 ± 15.4	34.2–34.5	10.0 ± 9.4	9.8–10.3	33.5 ± 7.4	33.4–33.6	<.001^∗^
Postoperative	23.4 ± 11.0	23.3–23.6	8.3 ± 7.9	8.1–8.5	24.8 ± 6.7	24.7–24.9	.001^∗^
Thoracic rotation (degrees)							
Pre-operative	38.7 ± 14.6	38.5–38.9	10.1 ± 7.4	9.9–10.2	44.9 ± 12.8	44.7–45.2	<.001^∗^
Postoperative	33.9 ± 16.6	33.7–34.1	9.1 ± 8.5	8.9–9.3	37.2 ± 9.5	37.0–37.3	.001^∗^

All parameters associated with sagittal depth and sternum deviation decreased significantly after surgery in the T-C group (all *P* < .001). All parameters also decreased significantly in the T-B group (all *P* < .01). The midline deviation decreased significantly (*P* = .034) after surgery, whereas the other measurements did not significantly differ between the pre-operative and postoperative values in the L-C group.

### 3.4 Surgical information and plain radiographic spinal deformities

The L-C group had a shorter operative time and fewer fused vertebrae than the other groups. Blood loss and usage of cell saver were not significantly different among the 3 groups.

A comparison of the radiographic parameters among the 3 groups is shown in Table 4. The T-C and T-B groups had greater Cobb angles of the main thoracic curve than the L-C group pre-operatively. However, the Cobb angles were not significantly different among the 3 groups postoperatively. The T-C and T-B groups had greater AVTs than the L-C group. Other X-ray parameters were not significantly different among the 3 groups.

**Table 4:** Surgical information and plain radiograph measurements of coronal and sagittal spinal parameters.

	T-C group (n = 28)		L-C group (n = 8)		T-B group (n = 13)		*P* value
		95% CI		95% CI		95% CI	
Operative time (min)	336.1 ± 88.4	335.0–337.1	213.1 ± 26.9	212.5–213.7	363.5 ± 69.7	362.3–364.8	<.001^∗^
Blood loss (mL)	914.3 ± 448.7	808.9–819.7	540.1 ± 216.8	535.1–545.1	684.6 ± 403.5	677.4–691.8	.186^∗^
Cell saver (%)	27 (96.4)		7 (87.5)		12 (92.3)		.625^†^
Number of fused vertebrae	10.4 ± 1.8	10.4–10.4	6.0 ± 0.9	6.0–6.0	9.2 ± 2.0	9.2–9.3	<.001^∗^
MT Cobb angle (°)							
Pre-operative standing	63.3 ± 17.3	63.1–63.5	31.1 ± 9.4	31.0–31.4	56.6 ± 10.1	56.5–56.8	<.001^∗^
Pre-operative traction	38.9 ± 16.4	38.7–39.1	17.9 ± 4.9	17.5–17.7	32.3 ± 13.4	32.0–32.5	<.001^∗^
Postoperative	22.1 ± 9.1	22.0–22.2	10.1 ± 18.9	18.7–19.1	21.9 ± 8.0	21.7–22.0	.960^∗^
TLL Cobb angle (°)							
Pre-operative standing	47.2 ± 15.3	47.0–47.4	47.3 ± 7.5	47.1–47.5	38.4 ± 10.4	38.2–38.6	.106^∗^
Pre-operative traction	26.0 ± 11.5	25.8–26.1	20.0 ± 5.5	19.9–20.1	20.5 ± 10.4	20.3–20.7	.200^∗^
Postoperative	17.9 ± 8.1	17.8–18.0	15.8 ± 4.7	15.6–15.9	21.7 ± 10.5	21.5–21.8	.586^∗^
MT AVT (°)							
Pre-operative standing	44.3 ± 19.9	44.0–44.5	9.3 ± 6.1	9.2–9.5	43.5 ± 11.3	43.3–43.7	<.001^∗^
Postoperative	16.8 ± 14.5	16.7–17.0	18.5 ± 12.7	18.2–18.7	11.0 ± 7.8	10.8–11.1	.392^∗^
Thoracic kyphosis (°)							
Pre-operative	23.4 ± 13.2	23.2–23.5	18.1 ± 8.3	17.9–18.3	15.0 ± 12.4	14.8–15.3	.094^∗^
Postoperative	19.1 ± 6.9	19.1–19.2	20.3 ± 7.8	20.2–20.5	17.3 ± 6.6	17.2–17.4	.785^∗^

Both the Cobb angles of the thoracic and thoracolumbar/lumbar main curves improved significantly in all groups (both curves in the T-C group, *P* < .001; both curves in the L-C group, *P* < .05; MT curve in the T-B group, *P* < .01; thoracolumbar/lumbar main curve in the T-B group, *P* < .05). AVT decreased after surgery in the T-C (*P* < .001) and T-B groups (*P* = .01) and increased in the L-C group (*P* = .036). Thoracic kyphosis was not significantly different before and after surgery in all groups (*P* = 0.093 in the T-C group, *P* = .050 in the L-C group, and *P* = .208 in the T-B group).

### 3.5 Case presentation with hypotension alarm during surgery

**Figure 6:**
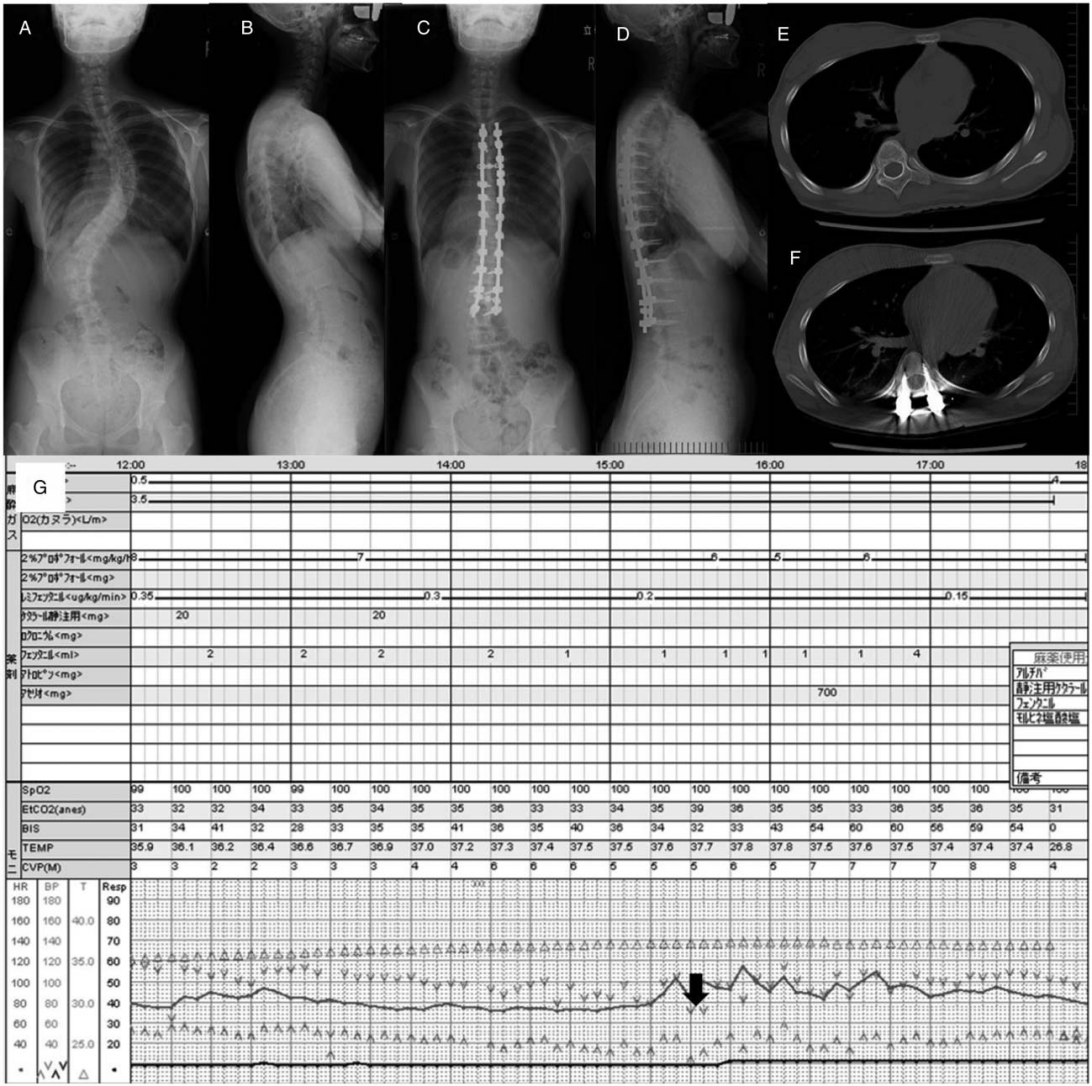
Case with hypotension alarm during surgery. A and B were the radiographs before surgery; C and D were after surgery. E and F were axial CT at the level of the apical vertebra before and after surgery. G was the record of the anesthesia and arterial pressure decreased at DVR procedure, shown by the black arrow. CT = computed tomography, DVR = direct vertebral rotation.

The patient, who was a 14-year-old female with adolescent idiopathic scoliosis, is shown in Figure 6. She underwent PSF using conventional rod attachment, RRM and DVR with 6.0 mm cobalt chrome rods. Mean arterial pressure decreased to 51.3 mm Hg at the timing of DVR. After the anesthesiologist informed surgeons about the hypotension, the DVR stopped promptly and hydration was performed by the anesthesiologist, then arterial pressure was elevated. The corrective procedure was resumed and there were no complications after surgery.

## 4 Discussion

We investigated the association between intra-operative hypotension and surgical procedures in adolescent patients with scoliosis, focusing on differences in curve patterns, corrective methods, and thoracic cage deformities. The rate of intra-operative alarm for hypotension was higher in patients with their main thoracic curve corrected using a 6.0-mm cobalt chrome rod. The corrective procedures associated with hypotension alarms were mounting rods, RRM, and DVR using a 6.0-mm cobalt-chromium alloy circular rod for correction of the main thoracic curve. Of the thoracic cage parameters, sagittal diameter and sternovertebral distance were not significantly different among the 3 groups, whereas the sternum deviation parameters, including midline deviation, thoracic rotation, and vertebral translation were greater in the T-C and T-B groups than those in the L-C group, both before and after surgery. In addition, both sagittal depth parameters, which were sagittal diameter and sternovertabral distance, and sternum deviation parameters decreased significantly after surgery in the T-C and T-B groups.

In this study, there were no patients with impaired hemodynamics when they were placed in the prone position. Brown et al^[15]^ prospectively investigated changes in the cardiac index and mean arterial pressure during scoliosis corrective surgery in the prone position and observed that the cardiac index decreased significantly from the supine to the prone position. A review of physiological changes in the prone position showed that the prone position can reduce cardiac output due to a reduction in preload when surgical patients are switched to the prone position during lumbar spine surgery.^[16]^ Therefore, hemodynamic changes should be carefully monitored when placing patients in the prone position during spinal deformity surgeries, although there was no record of such cases in our series.

Thoracic cage deformities were more severe in patients with main thoracic scoliosis; however, hypotension alarms occurred in the T-C group but not in the T-B group. Tauchi et al^[17]^ focused on the anteroposterior distance between the anterior thoracic wall and the spine in patients with scoliosis with pectus excavatum and observed some cases with a worse thoracic cage deformity after corrective surgery. In their series, no patients experienced hypotension during surgery. Neira et al^[18]^ described in a case report of a patient with scoliosis who had severe intra-operative hypotension in the prone position, and suggested that compression between the thoracic spine and the sternum produced positional obstruction of the right ventricular outflow tract with an increase in the right ventricular afterload and a decrease in the right ventricular compliance, which consequently led to an increase in the central venous pressure. Direct compression of the heart may not only result in compression of the right ventricle but also in myocardial ischemia, which leads to electrocardiogram abnormalities, such as partial right bundle-branch block.^[19]^ In this study, after only releasing correction, the blood pressure increased to normal levels in 3 out of the 7 patients with hypotension alarms. The anesthesiologists also determined that preload or/and cardiac output are needed to increase blood pressure; therefore, hydration and administering vasopressors were performed in the other 4 patients. The reason treatments other measures for treating hypotension, other than release of corrective procedures, were or not added may be associated with intra-operative blood loss; however, it is difficult to clarify the differences in the associated mechanisms in this study. We believe that although there are only a few critical hemodynamic changes in patients with thoracic cage deformities, corrective forces on the thoracic cage can narrow the anteroposterior diameter, and increase the risk of hypotension.

In the present study, rod attachment was the most frequent procedure associated with hypotension alarms, followed by DVR and RRM. In addition, as evaluation on CT, sagittal depth of thoracic cage decreased in both the T-C group and T-B group, and sternal deviation decreased too. Martino et al^[20]^ performed a biomechanical analysis of corrective techniques for thoracic scoliosis using a patient-specific model of the spine and observed that the first rod attachment and rod derotation maneuver induced regional corrections, such as Cobb angle correction, AVT, and thoracic kyphosis; the vertebral derotation maneuver mainly induced correction of apical vertebral rotation in the axial plane. Thoracic kyphosis is decreased by rod attachment and increased by the rod derotation maneuver, and this may be associated with geometric changes in the thoracic cage, which consequently leads to compression of organs during rod attachment. DVR added to simple rod derotation improved rotational correction compared to simple rod derotation^[21]^ and was useful for correcting rib deformities.^[22]^ Based on these reports, DVR was believed to influence both changes in spinal deformity in the axial plane and 3-dimensional thoracic cage deformity. Using a finite element method for analysis and the Cotrel-Dubousset method, Lafon et al^[23]^ investigated the kinematics of the thoracic scoliosis spine during corrective procedures. They reported that rod rotation of the concave side increased thoracic kyphosis and axial rotation and decreased the Cobb angle in the main thoracic curve. Changes in thoracic spinal deformity in the Cotrel-Dubousset method may be similar to that of rod rotation with all pedicle screw constructs. Lowenstein et al^[24]^ performed all screw correction with rod insertion, rod rotation, translation of the rod, distraction, and compression in adolescent patients with thoracic scoliosis and observed that postoperative thoracic kyphosis decreased significantly compared to pre-operative kyphosis. In the hybrid constructs, there was no difference between the pre-operative and postoperative thoracic kyphosis. Correction in the axial plane was better in all pedicle screw procedures than in the hybrid procedure because of DVR. This phenomenon might be associated with overgrowth of the thoracic vertebral body in cases of adolescent idiopathic scoliosis,[^25^,^26^] while Watanabe et al^[27]^ reported that thoracic kyphosis was reduced after complete correction of the coronal and rotational deformities using 3-dimensional simulation. From the standpoint of implant materials, cobalt chrome rods can achieve greater thoracic kyphosis postoperatively than stainless steel.^[28]^ Meanwhile, both hybrid^[29]^ and pedicle screw constructs^[30]^ were similar in sagittal correction using titanium and cobalt-chromium rods. From this study, thoracic scoliosis improved and anterior posterior diameter decreased in both the T-C and T-B groups, so these changes may occur regardless of type of rod and corrective methods. We should consider how these morphological, constructive, and material characteristics affect the sagittal plane during corrective surgery in patients with thoracic scoliosis. From previous reports and the results of the current study, hypotension during posterior spinal surgery for thoracic scoliosis may be associated with reduction of the thoracic cage antero-posterior diameter by corrective procedures.

To our knowledge, this study was the first report to clarify differences in occurrences of hypotension between several posterior corrective procedures in adolescent scoliosis patients. However, there were several limitations. First, this was a retrospective study and the results were obtained from analyzing the clinical data of patients who underwent surgeries by single surgeon. A prospective cohort involving multiple surgeons is needed to clarify the association between corrective procedures and intra-operative circulation and to generalize the results. Second, pre-operative severity might have differed between the T-C and T-B groups. This difference affected the statistical analyses; thus, further studies on patients with the same background, such as in a multicenter study involving more patients, is recommended. Third, we did not perform other direct measurements of arterial pressure such as ultrasound. Such an additional measurement might provide more information on dynamic changes during corrections and should be further investigated. Finally, this study had a small sample size. To achieve 80% statistical power with an alpha of 0.05, power analysis revealed that a minimum 27 participants would be required to detect any difference using chi-squared test. There was a significant difference in the occurrence of hypotension between the groups according to the curve patterns and implant types; however, further studies are required to show higher evidence. We believe that this study shows that the risk of hemodynamic change should be considered during corrective surgery for patients with thoracic scoliosis.

The following should be considered in PSF for adolescent scoliosis patients: the occurrence rate of intra-operative hypotension associated with corrective procedures is higher in thoracic scoliosis than in thoracolumbar/lumbar scoliosis; rod attachment may move the vertebral body to the ventral side and cause hypotension; RRM and DVR may increase thoracic kyphosis, but they may cause hypotension; and translational corrective procedures for thoracic scoliosis might prevent hypotension. Therefore, corrective procedures for thoracic constructive curve that require compressive force in the forward direction should be closely monitored as they could influence circulatory conditions. When mounting rods during constructive thoracic curve procedures, it may be better to gradually tighten the fixator in order to avoid forward compression.

## 5 Conclusions

This study demonstrated that decrease of blood pressure might be caused by cantilever forces during rod attachment, translation of vertebra at RRM, and compression of the rib cage at DVR in PSF for adolescent scoliosis patients with thoracic curve. Sagittal depth parameters, such as sagittal diameter and sternovertebral distance, decreased after PSF in patients with thoracic curve, and the parameters did not change in thoracolumbar/lumbar scoliosis patients. Furthermore, sternum deviation parameters, such as midline deviation, vertebral translation and thoracic rotation, decreased significantly after PSF for thoracic scoliosis, compared to thoracolumbar/lumbar scoliosis.

## Acknowledgments

The authors would like to thank Prof. Kazuyoshi Hirota and staff of the Department of Anesthesiology, Hirosaki University Graduate School of Medicine for their valuable advice and the record of intra-operative data. We would like to thank Editage (www.editage.jp) for the English language editing.

## Author contributions

**Conceptualization:** Kanichiro Wada.

**Data curation:** Kanichiro Wada.

**Formal analysis:** Kanichiro Wada.

**Investigation:** Kanichiro Wada, Gentaro Kumagai, Hitoshi Kudo, Sunao Tanaka, Toru Asari, Yuki Fjita.

**Supervision:** Yasuyuki Ishibashi.

**Writing – original draft:** Kanichiro Wada.

**Writing – review & editing:** Gentaro Kumagai, Hitoshi Kudo, Sunao Tanaka, Toru Asari, Yasuyuki Ishibashi.
